# Supplementary material for: Myostatin regulates fatty acid desaturation and fat deposition through MEF2C/miR222/SCD5 cascade in pigs
Source: Commun Biol. 2020 Oct 23;3:612. doi: 10.1038/s42003-020-01348-8 (PMC7584575; doi:10.1038/s42003-020-01348-8)
Supplement: Supplementary file 1 — Supplementary Information [file 42003_2020_1348_MOESM1_ESM.pdf]

1、 Supplementary Figures

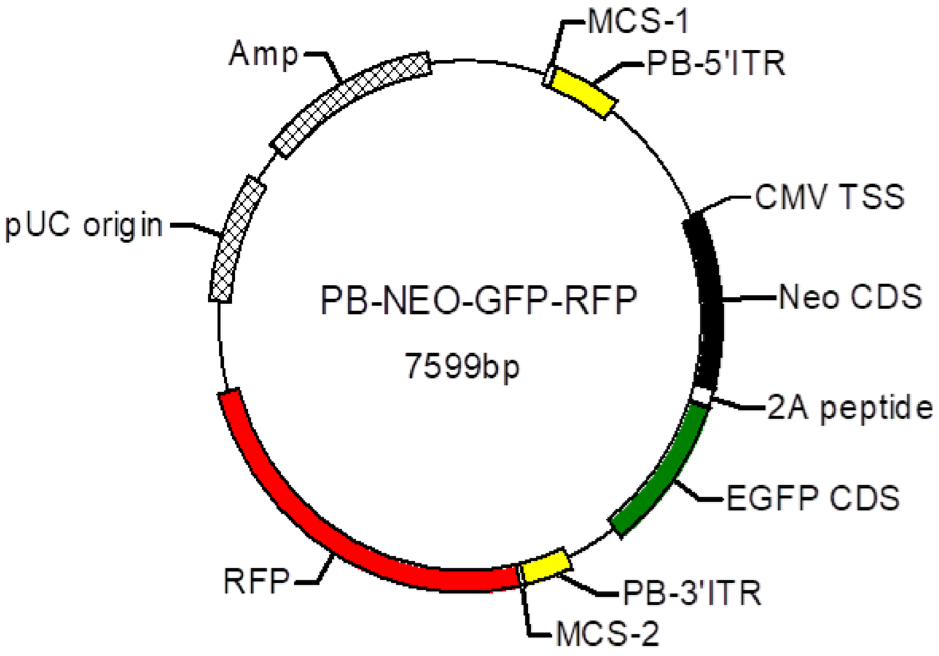

Supplementary Fig. 1. Schematic of targeting plasmid backbone PB-NEO-GFP-RFP

PB-5´ITR and PB-3´ITR represent the 5´ and 3´ inverted tandem repeats of Piggybac transposase. CMV TSS, transcription start site of CMV promoter. MCS-1 and MCS-2 represent multiple cloning sites.

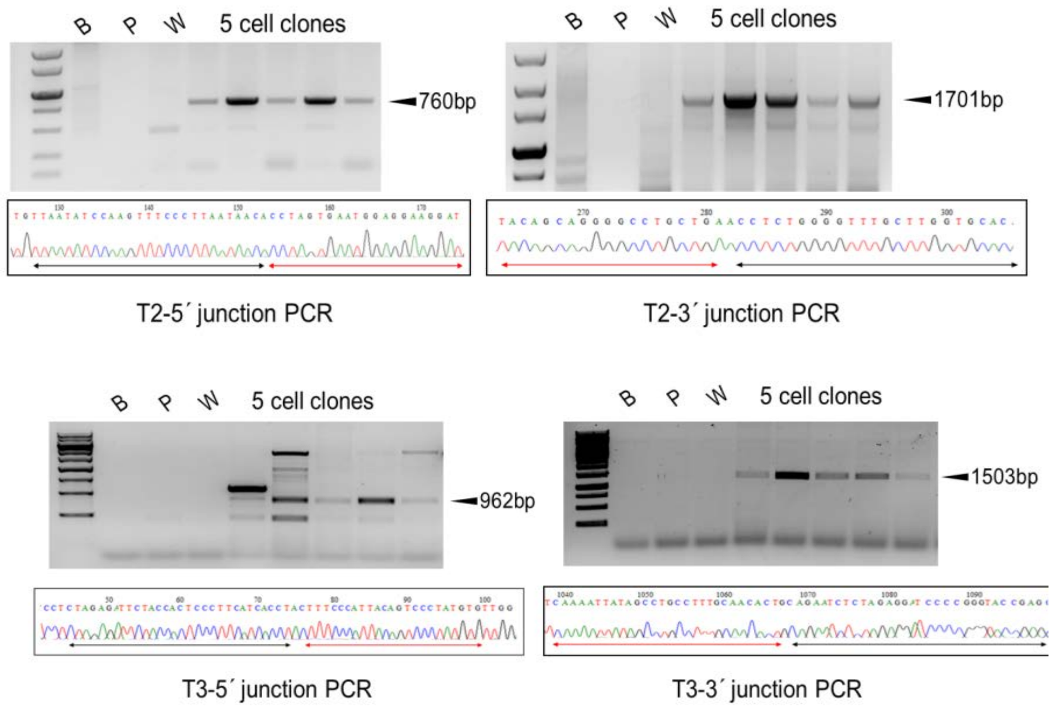

Supplementary Fig. 2. Representative image of DUFAS-mediated MSTN targeting at T2 and T3 sites

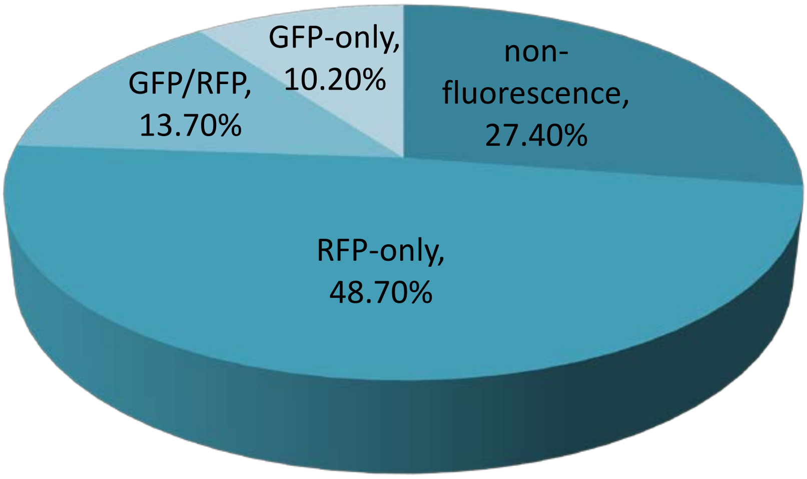

Supplementary Fig. 3 Distribution of cell clones with distinctive fluorescence post Neomycin selection

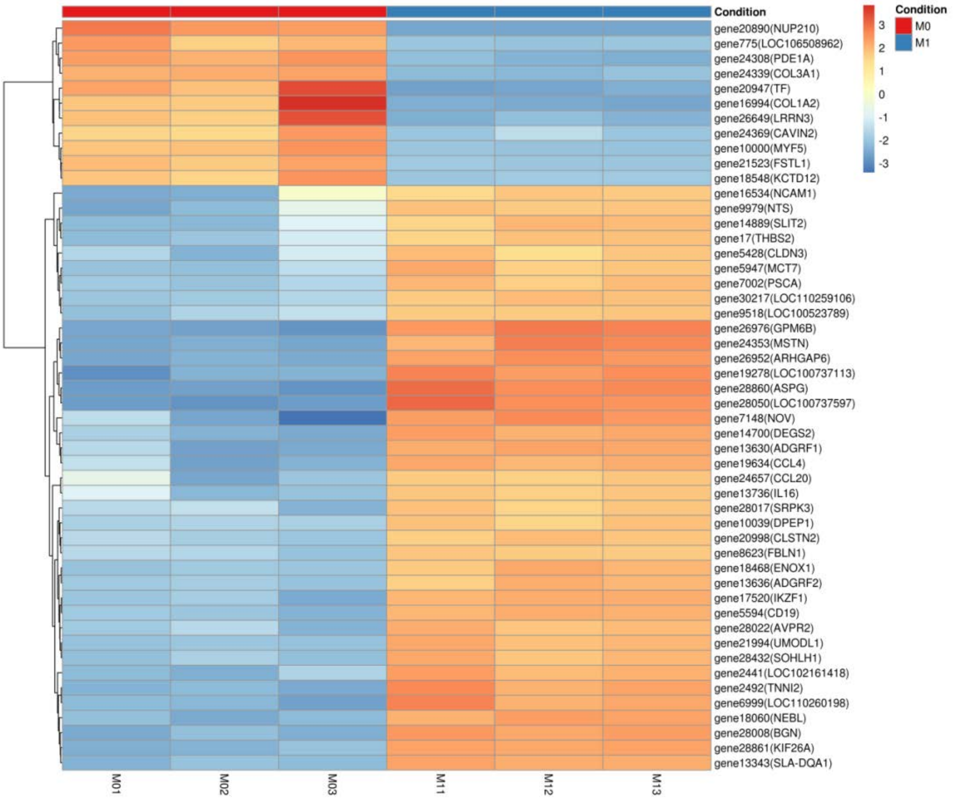

Supplementary Fig. 4. Heatmap of top 50 DE mRNAs

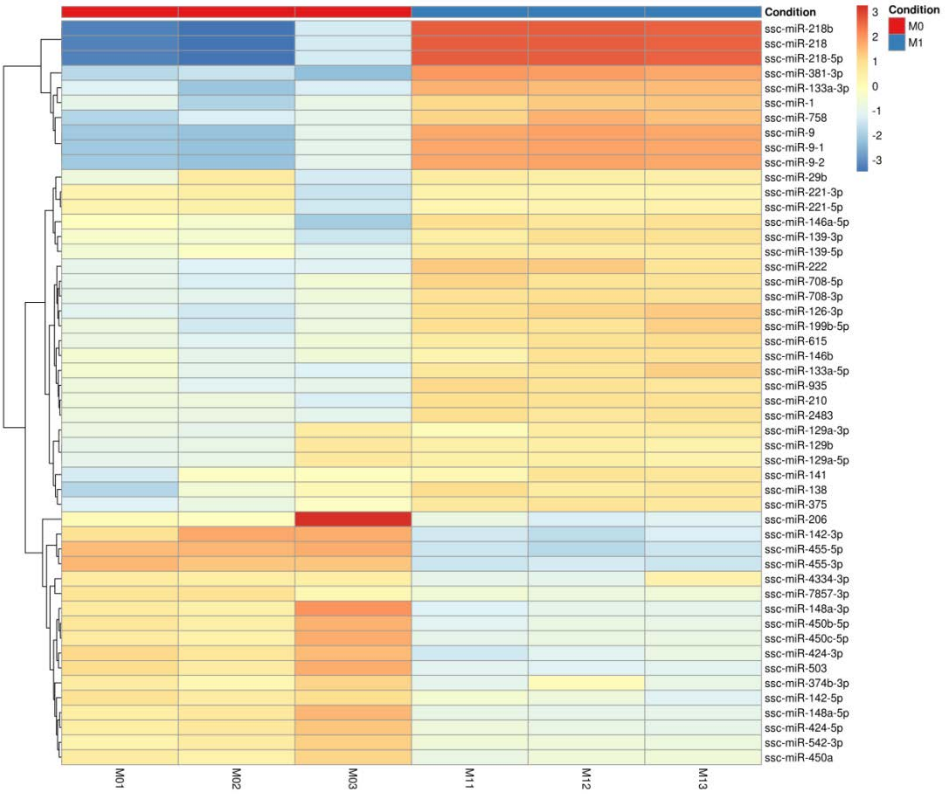

Supplementary Fig. 5. Heatmap of top 50 DE miRNAs

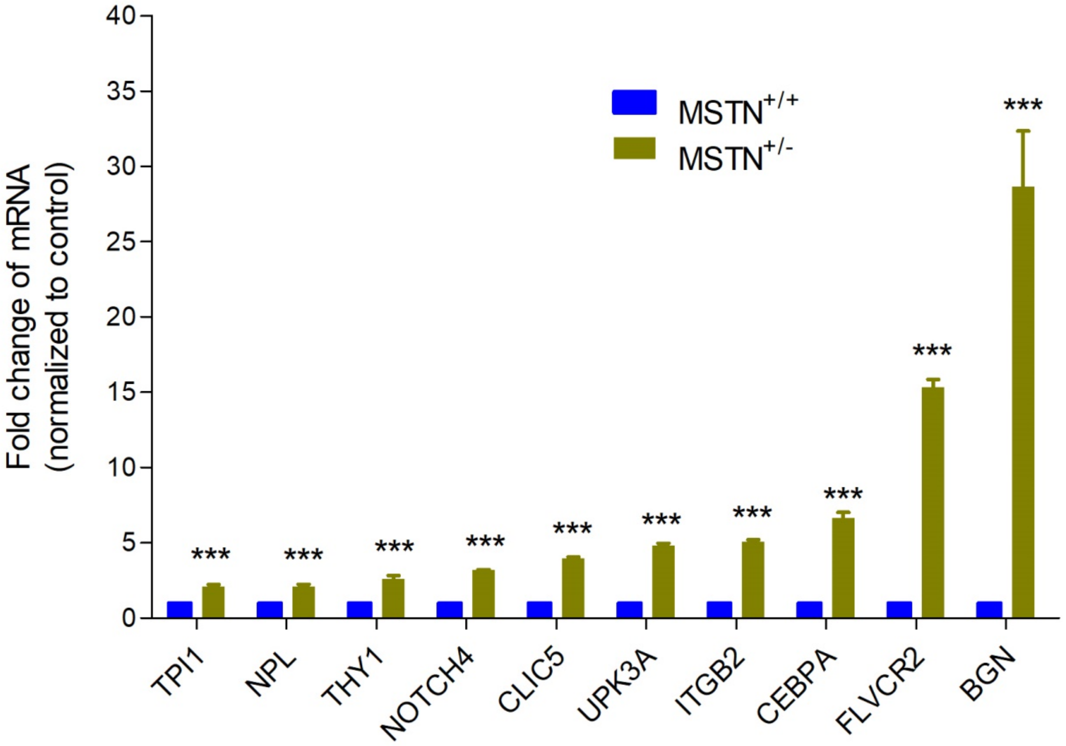

Supplementary Fig. 6. Q-PCR validation of the expression of 10 up-regulated mRNAs

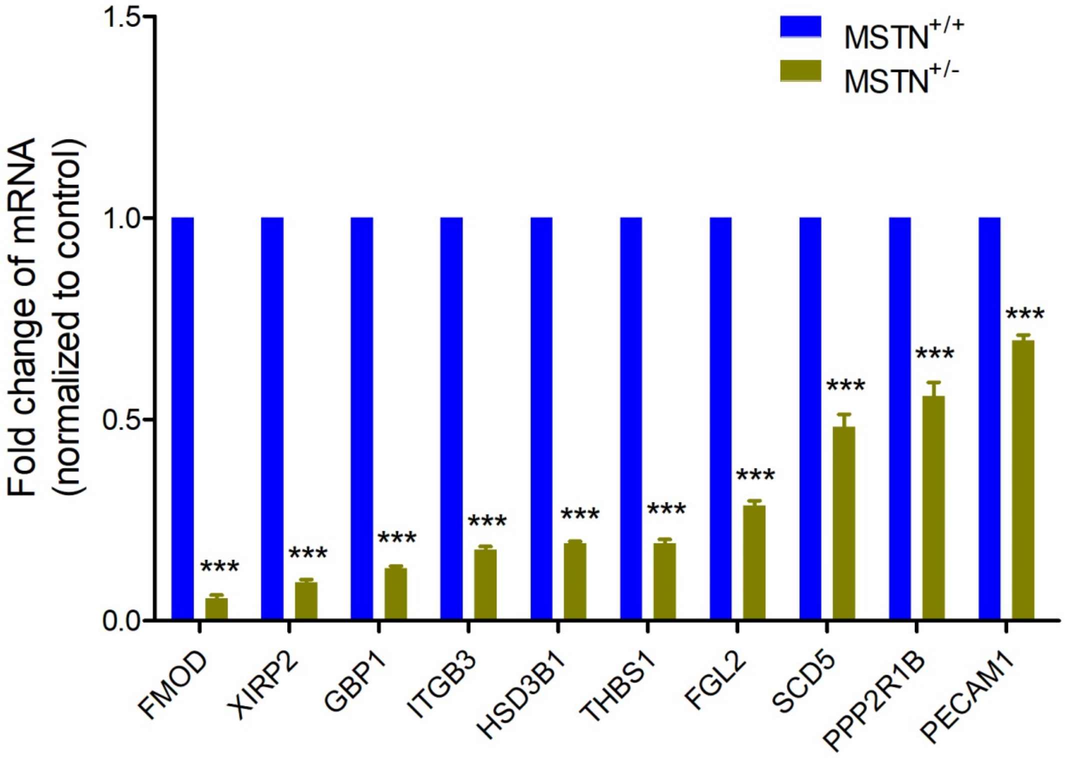

Supplementary Fig. 7. Q-PCR validation of the expression of 10 down-regulated mRNAs

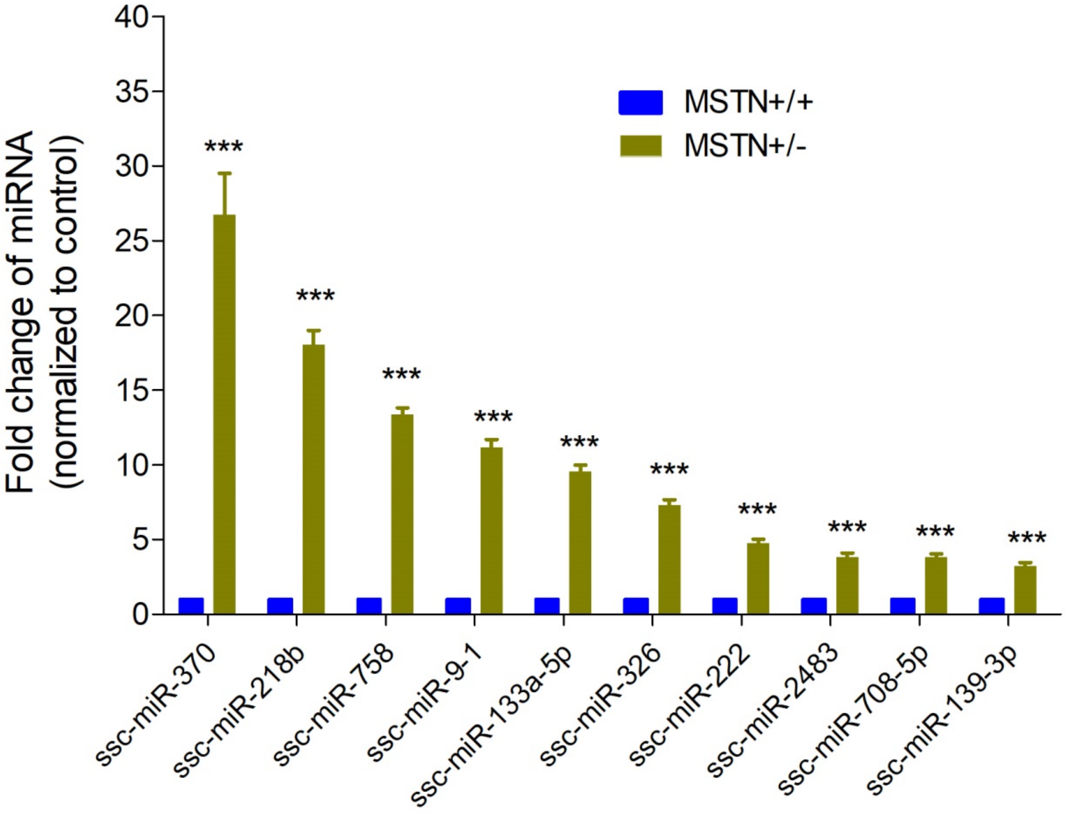

Supplementary Fig. 8. Q-PCR validation of the expression of 10 up-regulated miRNAs

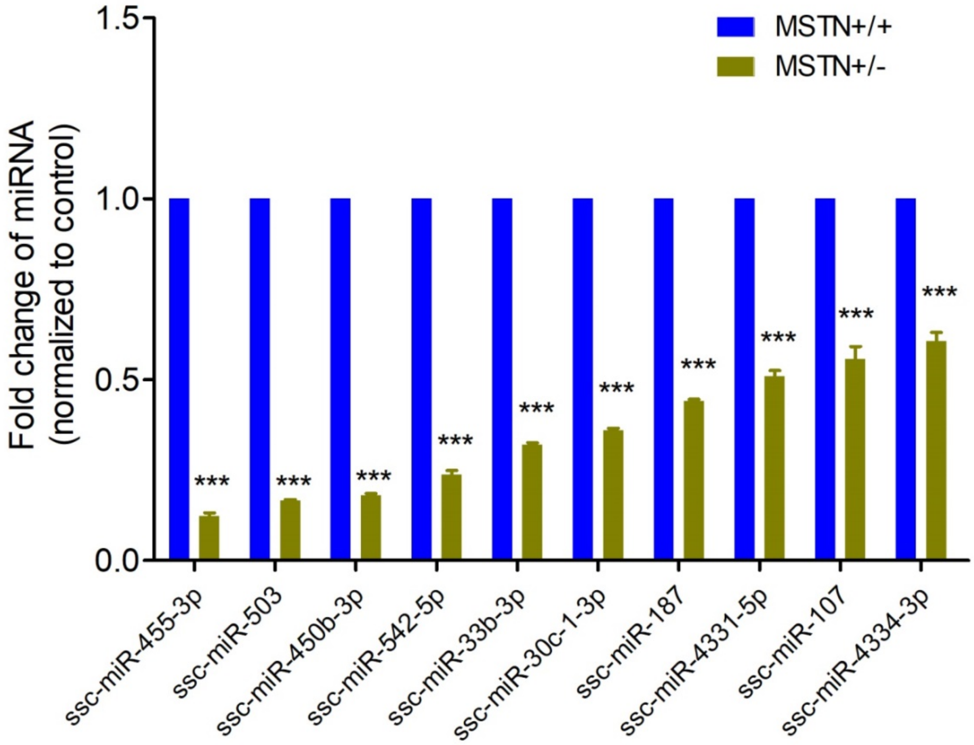

Supplementary Fig. 9. Q-PCR validation of the expression of 10 down-regulated miRNAs

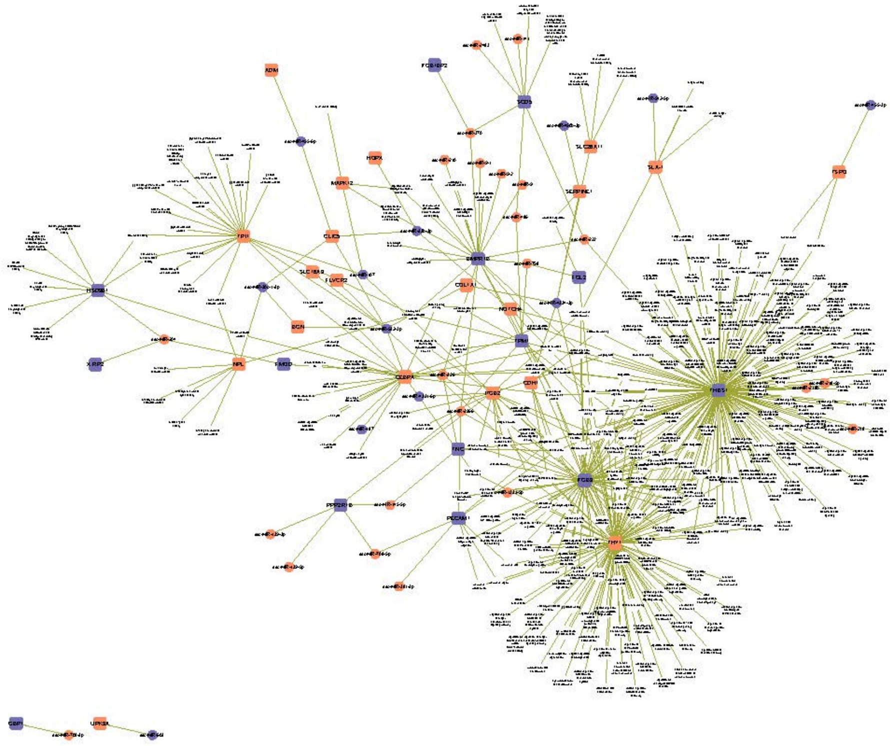

Supplementary Fig. 10. DE miRNA-mRNA network

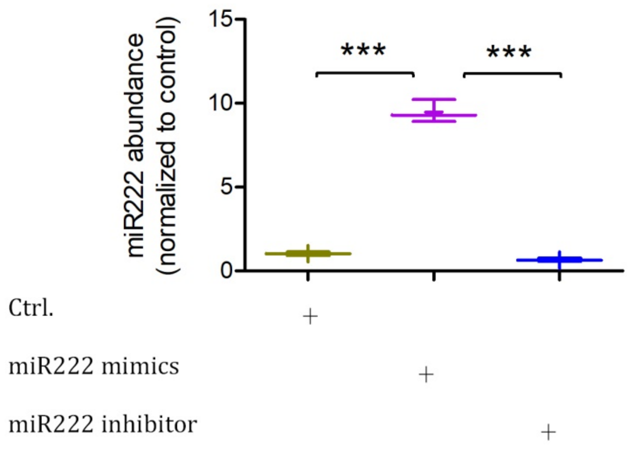

Supplementary Fig. 11. miR222 abundance with the treatment of miR222 mimics and inhibitor

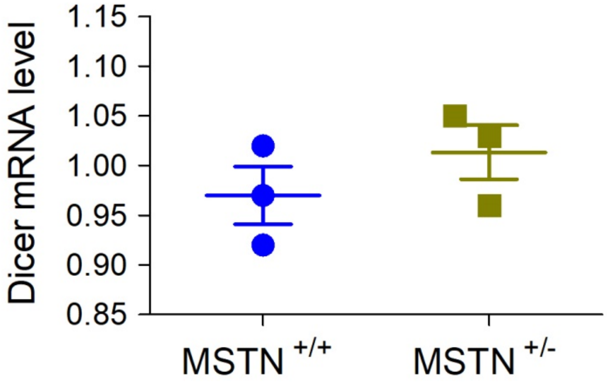

Supplementary Fig. 12. Dicer mRNA abundance in MSTN KO and wild-type pigs

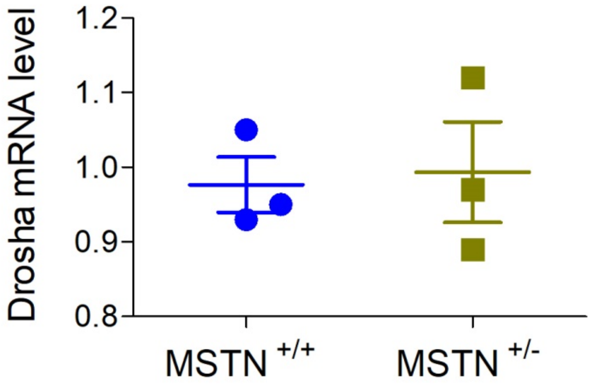

Supplementary Fig. 13. Drosha mRNA abundance in MSTN KO and wild-type pigs

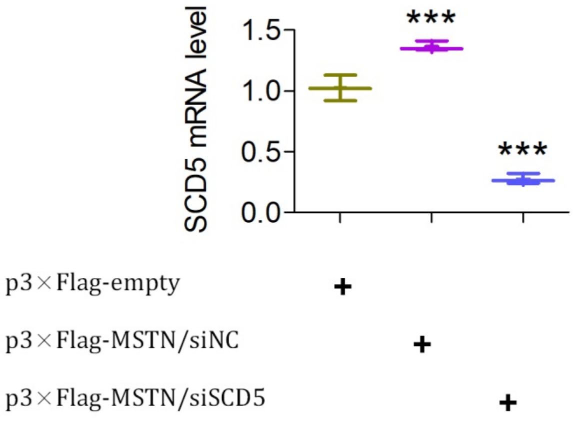

Supplementary Fig. 14. SCD5 mRNA abundance after treatment of siRNA

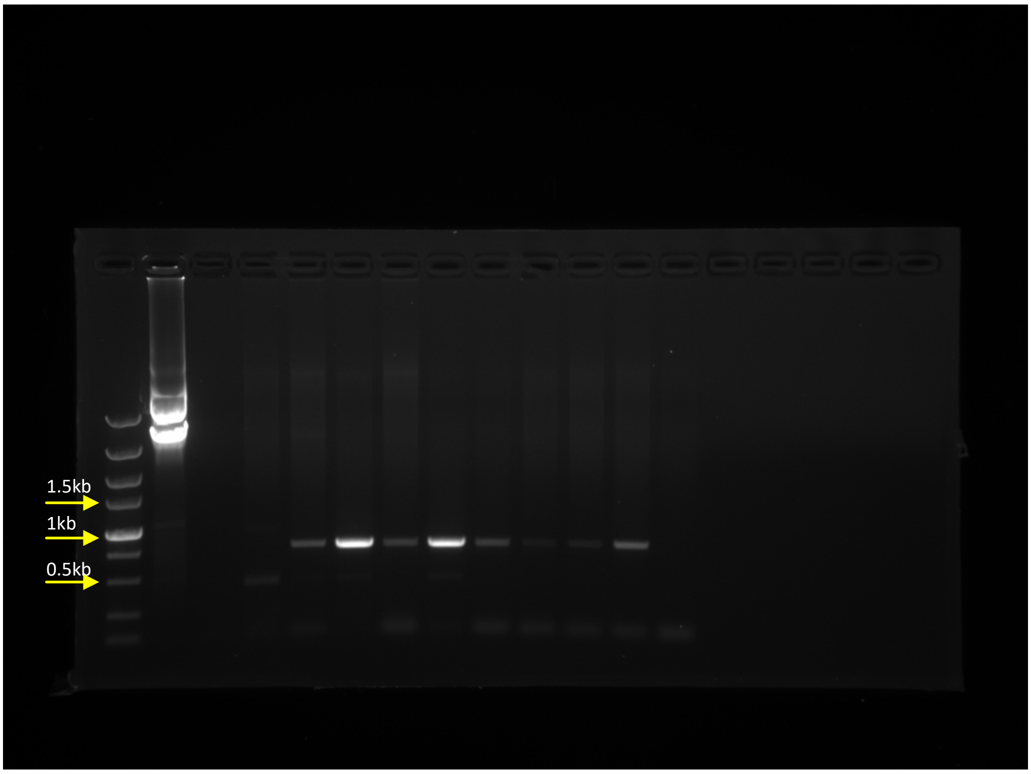

Supplementary Fig. 15 Uncropped gel image of junction PCR

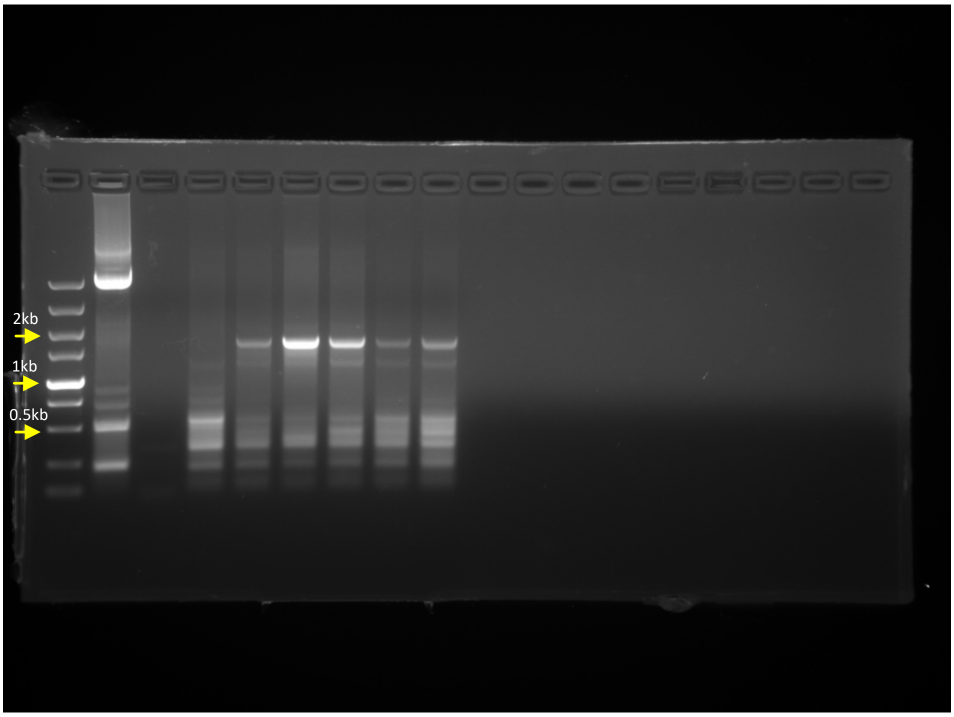

Supplementary Fig. 16 Uncropped gel image of junction PCR

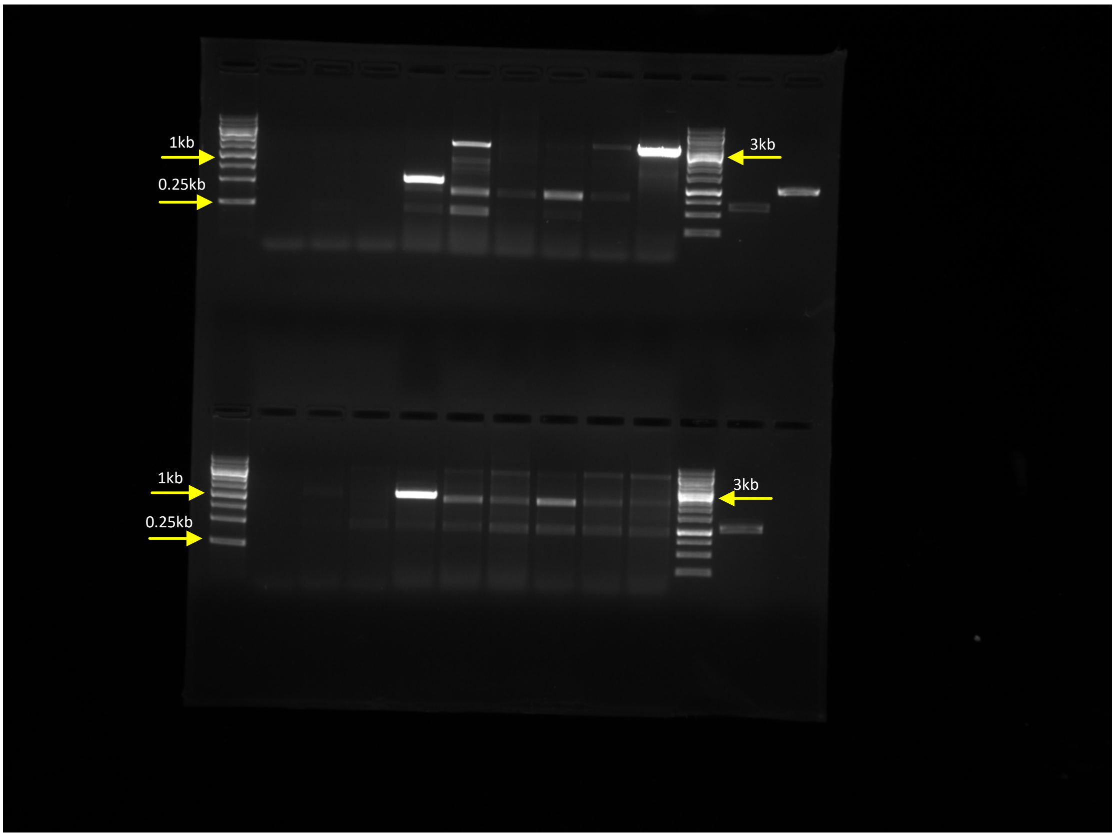

Supplementary Fig. 17 Uncropped gel image of junction PCR

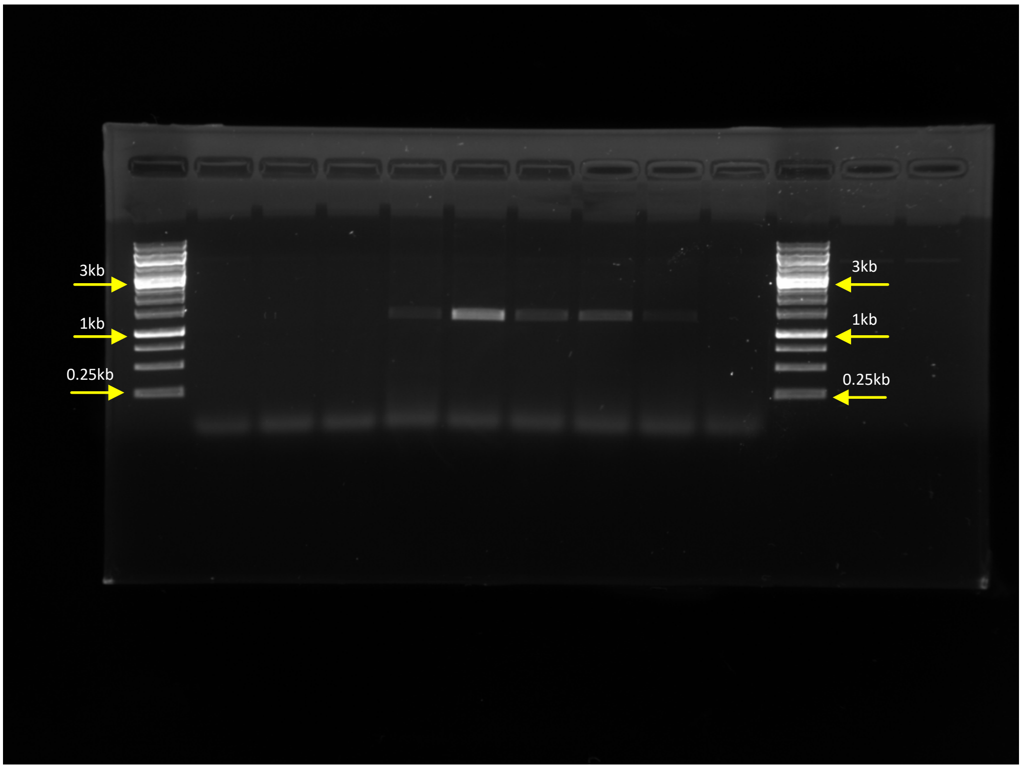

Supplementary Fig. 18 Uncropped gel image of junction PCR

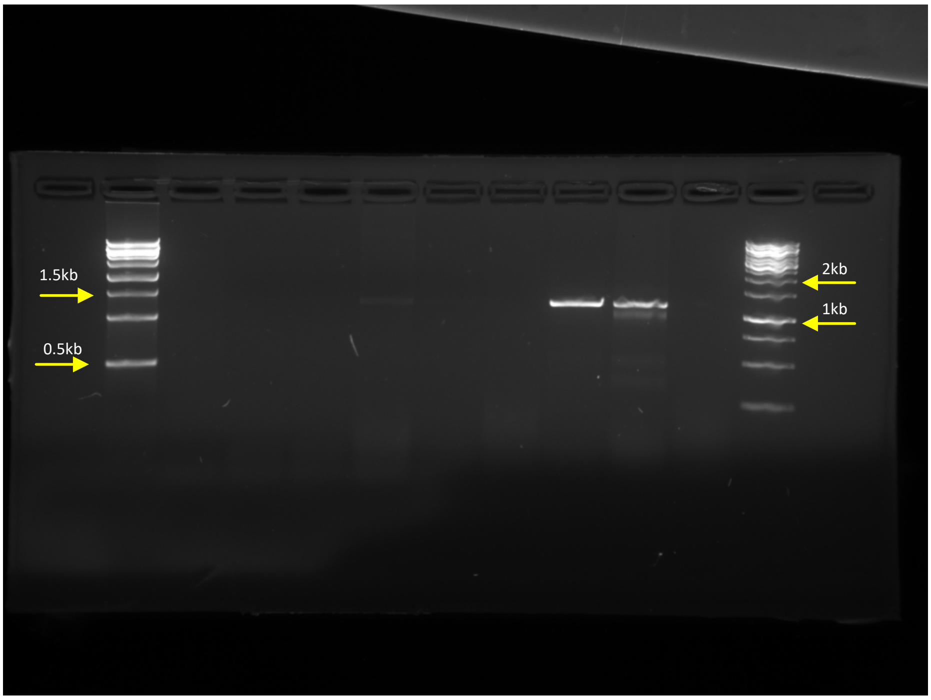

Supplementary Fig. 19 Uncropped gel image of junction PCR

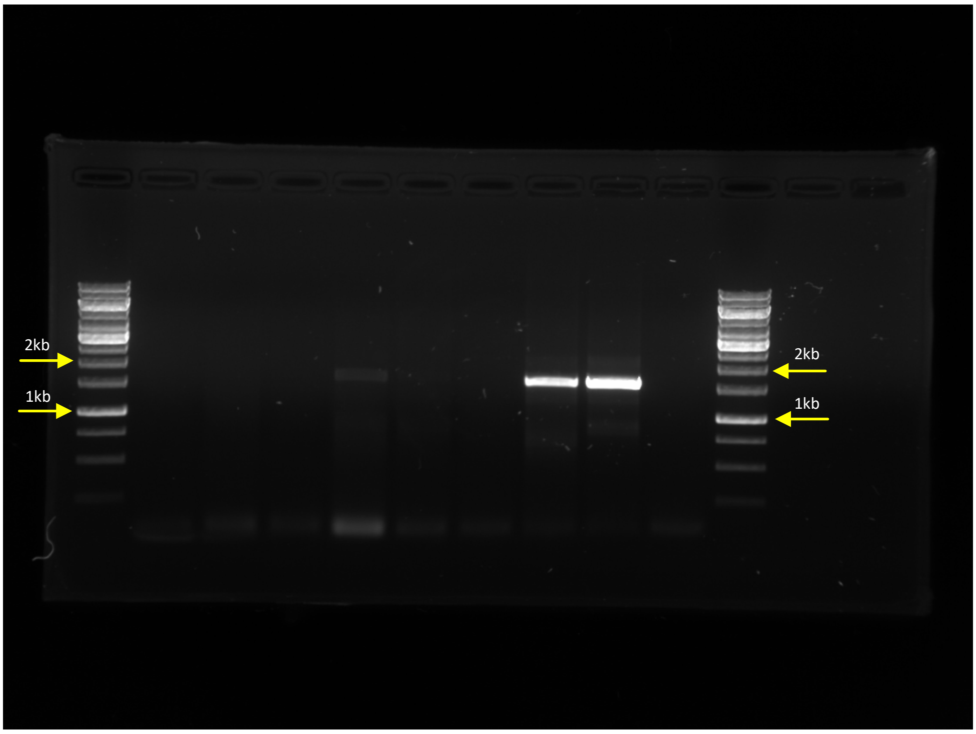

Supplementary Fig. 20 Uncropped gel image of junction PCR

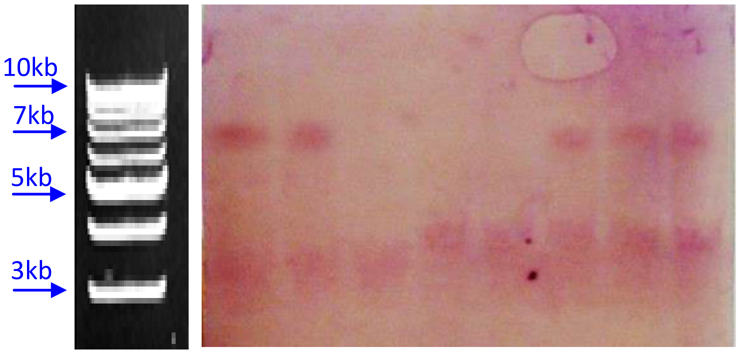

Supplementary Fig. 21 Uncropped blot image of Southern blotting in Fig. 1f

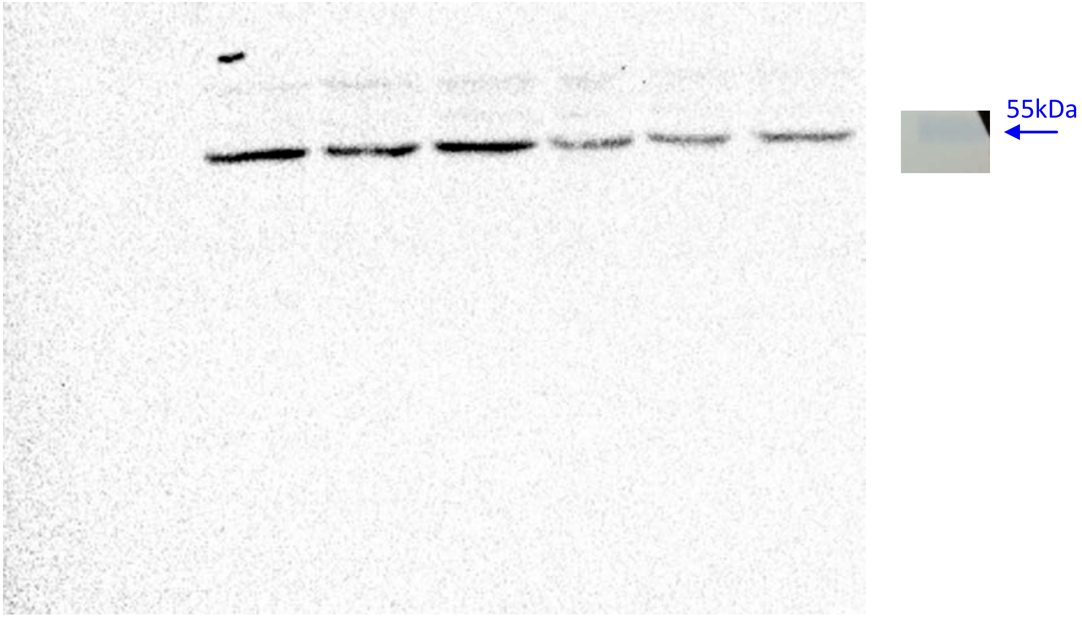

Supplementary Fig. 22 Uncropped blot image of Western blotting for MSTN in Fig. 1l

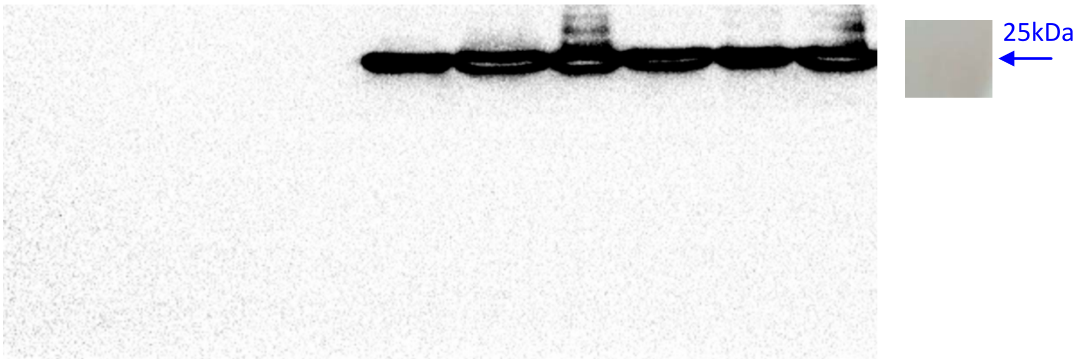

Supplementary Fig. 23 Uncropped blot image of Western blotting for GAPDH in Fig. 1l

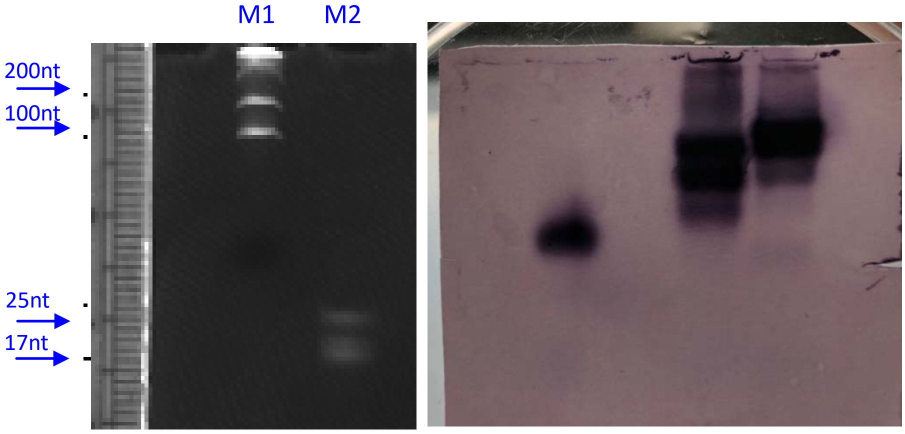

Supplementary Fig. 24 Uncropped blot image of Northern blotting for miR222 in Fig. 2h

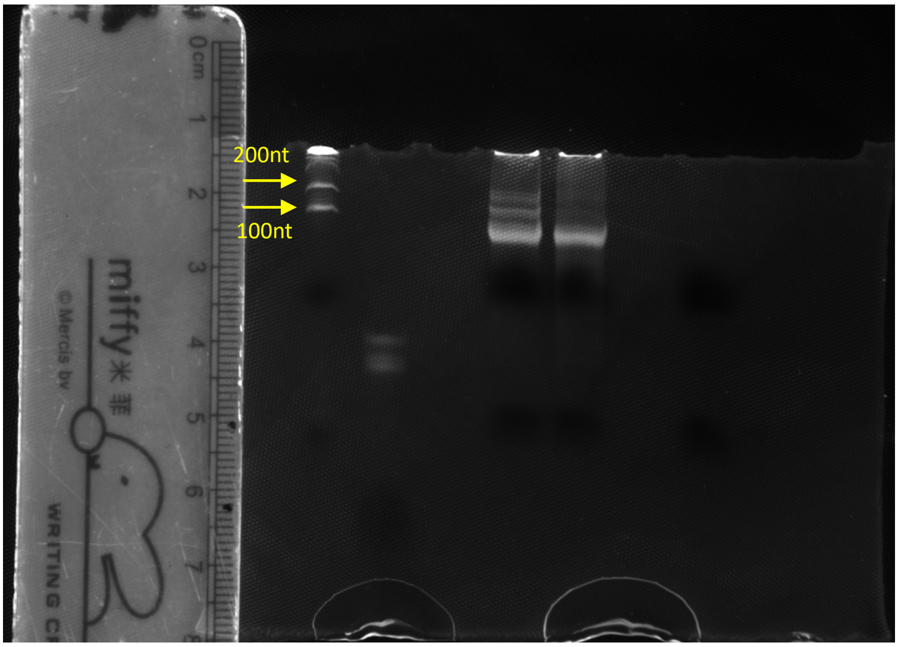

Supplementary Fig. 25 Uncropped gel image of PAGE for small RNA in Fig. 2h

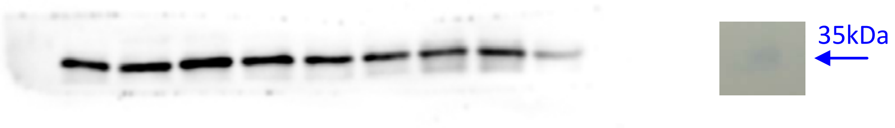

Supplementary Fig. 26 Uncropped blot image of Western blotting for SCD5 Fig. 2j

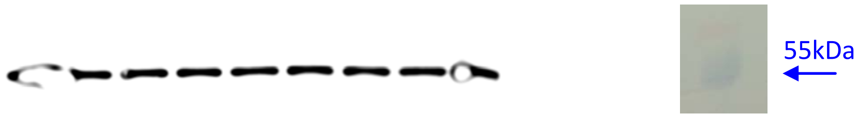

Supplementary Fig. 27 Uncropped blot image of Western blotting for tubulin Fig. 2j

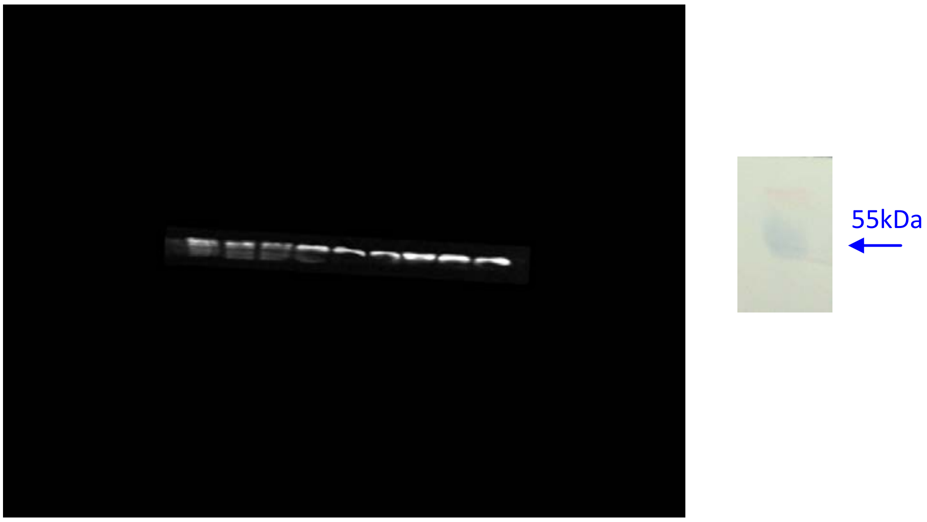

Supplementary Fig. 28 Uncropped blot image of Western blotting for MEF2C Fig. 4b

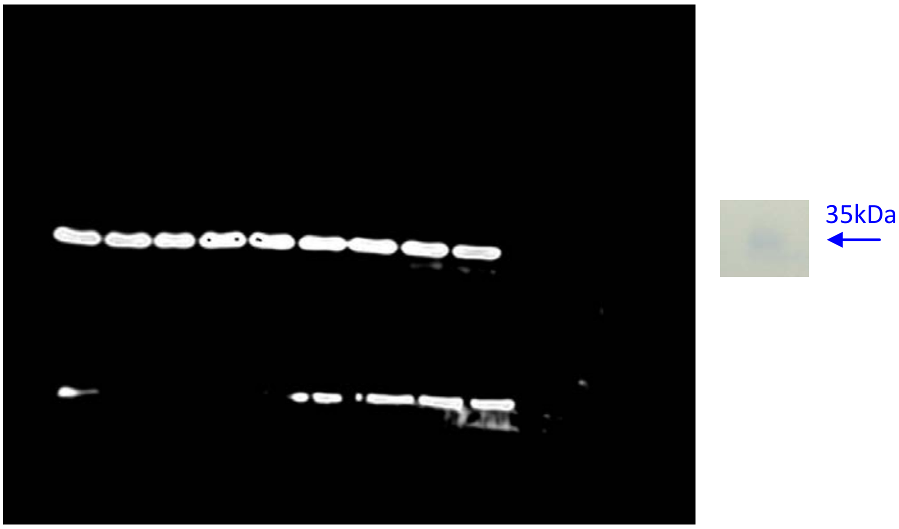

Supplementary Fig. 29 Uncropped blot image of Western blotting for GAPDH Fig. 4b

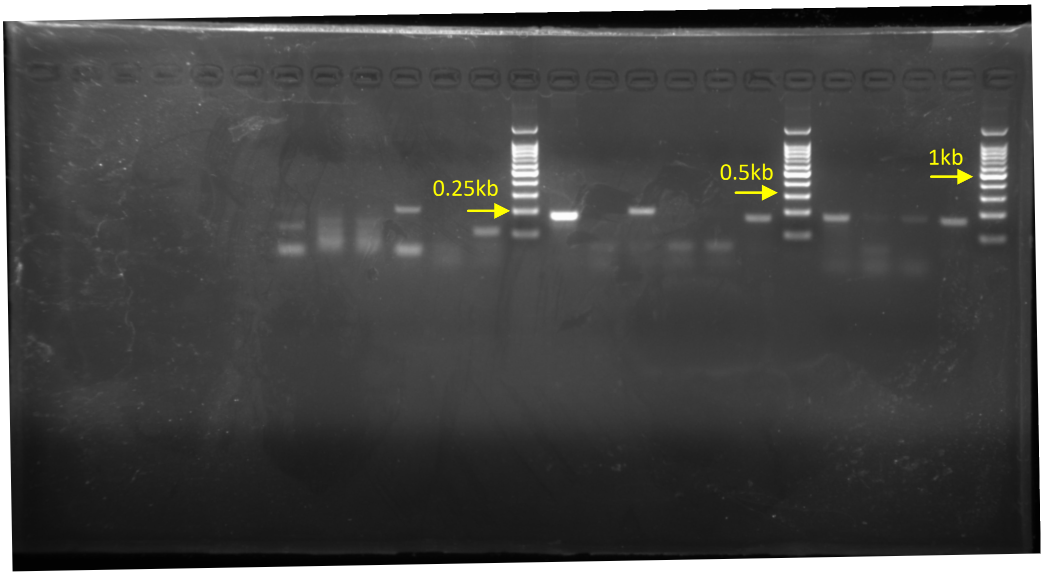

Supplementary Fig. 30 Uncropped gel image of ChIP-PCR in Fig. 4h

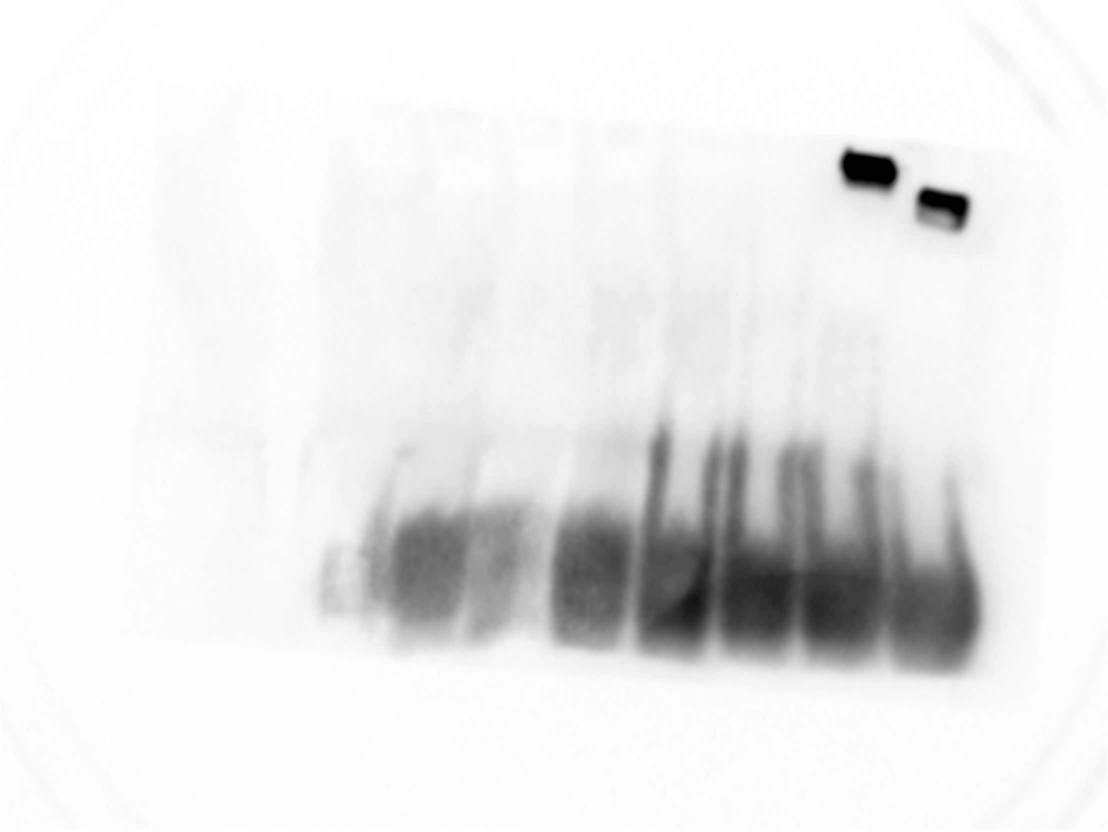

Supplementary Fig. 31 Uncropped gel image of EMSA in Fig. 4k

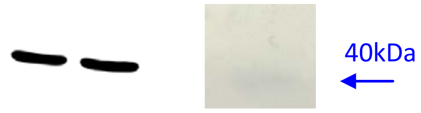

Supplementary Fig. 32 Uncropped blot image of Flag-MSTN in Fig. 5a

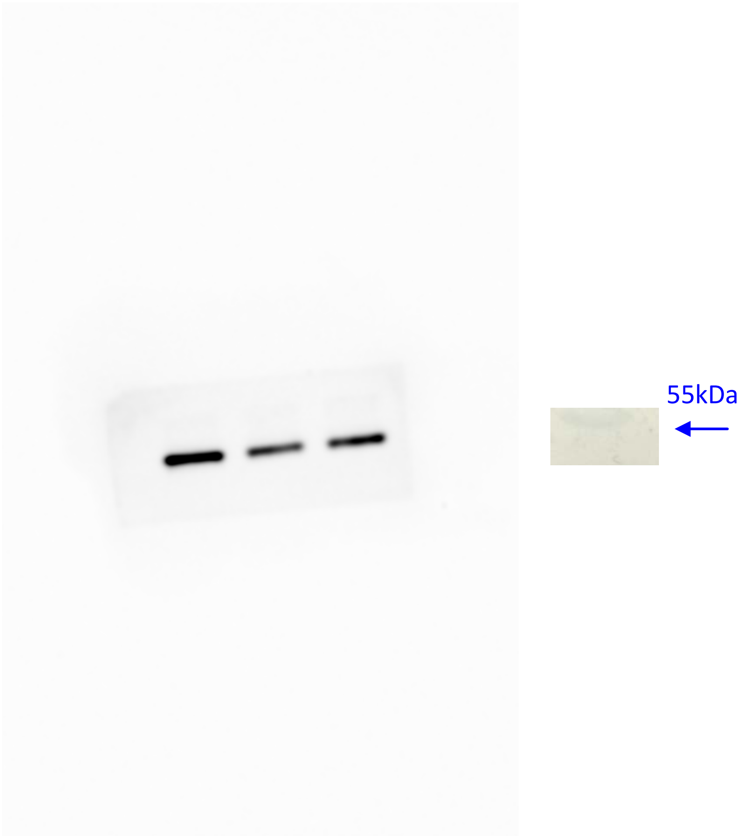

Supplementary Fig. 33 Uncropped blot image of MEF2C in Fig. 5a

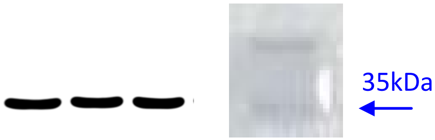

Supplementary Fig. 34 Uncropped blot image of GAPDH in Fig. 5a

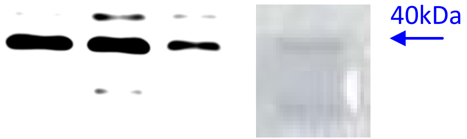

Supplementary Fig. 35 Uncropped blot image of SCD5 in Fig. 5a

2、 Supplementary Tables

Supplementary Table 1. DUFAS-mediated HDR efficiency at multiple sites in immortal and primary porcine cells

Exp.	Cell type	Gene	Target region	CRISPR editing site	Aim	Deletion	Insertion	EGFP-only clones (No.)	HDR clones (No.)	HDR efficiency
0$	Porcine PK15	MSTN	E3	T3	KO	381 bp	3 kb Neo^R/EGFP cassette	32	2	6.3%
1	Porcine PK15	MSTN	E1	T1	KO	0 bp	3 kb Neo^R/EGFP cassette	36	29	80.5%
2	Porcine PK15	MSTN	E3	T2	KO	0 bp	3 kb Neo^R/EGFP cassette	30	26	86.7%
3	Porcine PK15	MSTN	E3	T3	KO	0 bp	3 kb Neo^R/EGFP cassette	29	24	82.3%
4	Meishan pig primary fibroblasts	MSTN	E1	T1	KO	0 bp	3 kb Neo^R/EGFP cassette	17	14	82.3%

$Experiment from reference 37. T1 targeting site, located in exon 1 of porcine MSTN, sequence 5´GGCTGTGTAATGCATGTATG3´; T2 targeting site, located in exon 3 of porcine MSTN, sequence 5´GGATTTTGAAGCTTTTGGAT3´; T3 targeting site, located in exon 3 of porcine MSTN, sequence 5´GGCAAAGAACAAATAATATA3´. KO, knockout. Exp., experiment.

Supplementary Table 2. SCNT results using primary fetal fibroblast cells as donor nuclei

Cloning date	Recipient	Donor cells	Cleavage rate (%)	No. of transferred reconstructed embryos	Pregnant sows	Live born	Cloning efficiency (%)	Piglet number and birth weight (kg)
5/18/2018	#20304	#MS30	68.25	167	aborted	-	-	
5/19/2018	#1711	#MS30	70.13	161	Delivered	4	2.48	#1020(0.83), #1021(1.15), #1022(0.96), #1023(1.31)

Supplementary Table 3. Profile of mRNA sequencing

Sample ID.	Clean reads	Mapped reads	Uniquely mapped reads	Paired mapped reads	Unmapped reads	Transcript coverage (x)
M01	75247130	73073340	68410273	35660525	2173790	123.48
M02	64740926	62810918	59133062	30595861	1930008	108.92
M03	71910194	69371613	65079360	33667011	2538581	116.55
M11	63346590	60989110	56914156	29592064	2357480	102.75
M12	65214872	62878188	58491445	30552730	2336684	105.51
M13	65018918	62780972	58425018	30498874	2237946	105.42

Supplementary Table 4. Profile of small RNA sequencing

Sample ID.	Clean reads	miRNA	rRNA	snoRNA	Mapped reads	Unmapped reads
M01	10563627	245	144	2525	8612509	1951118
M02	12705181	235	152	3315	10750354	1954827
M03	8602572	244	123	2698	7318434	1284138
M11	7762315	243	97	2090	5961153	1801162
M12	8458847	249	39	245	5790788	2668059
M13	10806313	253	63	398	7212217	3594096

Supplementary Table 5. Prediction and filtering result of transcription factors (TF) on miR222 promoter

TF	Binding site	Dissimilarity	log_2FoldChange in transcriptome
MEF2C	TATAAATACTT(A), GTGTGTGTATA(B)	3.69	2.46670082842888
IRF4	TTTCCCTAC	4.39	1.64628583932162
CEBPB	TGCAAT	0.00	1.20156404677452
JUN	AGAGTCA	3.24	1.23961703695867
EP300	AGTCC	0.00	-1.268671954
MEF2A	TATTTATAGCT	2.66	-1.11513398752536
