# Supplementary material for: Myostatin regulates fatty acid desaturation and fat deposition through MEF2C/miR222/SCD5 cascade in pigs
Source: Commun Biol. 2020 Oct 23;3:612. doi: 10.1038/s42003-020-01348-8 (PMC7584575; doi:10.1038/s42003-020-01348-8)
Supplement: Supplementary file 3 — Description of additional supplementary files [file 42003_2020_1348_MOESM3_ESM.docx]

Description of Additional Supplementary Files

Supplementary Data 1: Source data underlying plots shown in figures.

Supplementary Data 2: Primer sequences.
