# Supplementary material for: Myostatin regulates fatty acid desaturation and fat deposition through MEF2C/miR222/SCD5 cascade in pigs
Source: Commun Biol. 2020 Oct 23;3:612. doi: 10.1038/s42003-020-01348-8 (PMC7584575; doi:10.1038/s42003-020-01348-8)
Supplement: Supplementary file 5 — Supplementary Data 2 [file 42003_2020_1348_MOESM5_ESM.doc]

Supplementary Data 2

Table 1. Oligos used in this study

| **Name** | **Sequence** | **Comments** |
| --- | --- | --- |
| 2A-F1 | agca CTCGAG AAAATTGTCGCTCCTGTC (XhoI) | To generate the DNA sequence for 2A peptide |
| 2A-R1 | acga GGATCC TGGACCTGGATTGCTTTC (BamHI) |
| Lox19-F1 | acga GGATCC ATGGTGAGCAAGGGCGAGG (BamHI) | To generate the backbone DNA sequence (removing NEO stop codon) in order to ligate the 2A sequence |
| Lox19-R1 | agca CTCGAG GAAGAACTCGTCAAGAAGG (XhoI) |
| DsRed-F3 | agct GGTACC CCTAGG CCGCGG CTCCCTTTAGGGTTCCG | DsRed cassette subcloning into the PB-NEO-EGFP-RFP backbone plasmid |
| DsRed-R2 | agct ACCGGT TAGTTATTAATAGTAATC |
| Cas9-T1-LF | gcag GAATTC GAGGAAAAAGACATTTCAAC (EcoRI) | Left homolog arm for T11 targeting site at MSTN locus, 612bp. It was also the probe for southern blotting in Meishan cloned pigs. |
| Cas9-T1-LR | gcag ATCGAT CACAGCCCCTCTTTTTCC (ClaI) |
| Cas9-T1-RF | cagc GTCGAC TGGAGACAAAACACTAAATC (SalI) | Right homolog arm for T11 targeting site at MSTN locus, 1009bp |
| Cas9-T1-RR | cacg atgcat TTAAGCATGTGCCTGCCTGG (NsiI) |
| Cas9-T2-LF | gcag GAATTC CCTAGTGAATGGAGGAAGG (EcoRI) | Left homolog arm for T23 targeting site at MSTN locus, 555bp |
| Cas9-T2-LR | gcag ATCGAT GCTTCAAAATCCACAGTTAG (ClaI) |
| Cas9-T2-RF | cagc GTCGAC ATGGGACTGGATTATTGC (SalI) | Right homolog arm for T23 targeting site at MSTN locus, 423bp |
| Cas9-T2-RR | cacg atgcat GGTTAAATGCCAACCATTGC (NsiI) |
| Cas9-T3-LF | gcag GAATTC CCTAGTGAATGGAGGAAGG (EcoRI) | Left homolog arm for T31 targeting site at MSTN locus, 758bp |
| Cas9-T3-LR | gcag ATCGAT TTATTTGTTCTTTGCCAT (ClaI) |
| Cas9-T3-RF | cagc GTCGAC ATATGGGAAAATTCCAGCC (SalI) | Right homolog arm for T31 targeting site at MSTN locus, 804bp |
| Cas9-T3-RR | cacg atgcat GCAGTTTCTCCAAGTATGC (NsiI) |
| T3-JK-F1 | GCCAACTAATTTATTCCAAGGCC | To use with MKR for the detection of 5´ junction of MSTN locus targeted at exon 3 |
| RA-down | GCCATGCTGTCATTGTAAGCAG | To use with EGFP-QF for the detection of 3´ junction of MSTN locus targeted at exon 3 |
| T1-UP | CTGGAAATCTGAGGCAAACTGC | To use with MKR for the detection of 5´ junction of MSTN locus at T11 site (KI) |
| T1-Down | GCCTTCTCTGTTAAGGTGCCTG | To use with EGFP-QF for the detection of 3´ junction of MSTN locus at T11 site (KI) |
| T2/3-UP | CTACCACTCCCTTCATCACCTAC | To use with MKR for the detection of 5´ junction of MSTN locus at T23/T31 site (KI) |
| T2/3-DOWN | CTGCAGTGTTGCAAAGGCAGGC | To use with EGFP-QF for the detection of 3´ junction of MSTN locus at T23/T31 site (KI) |
| 5-LTR-F | GCTA ATCGAT TTAACCCTAGAAAGATA GTC | To generate the 5´ LTR of Piggybac transposon element |
| 5-LTR-F | GCCG ATCGAT GATATCTATAACAAGAAAA |
| 3-LTR-F | ccgg GTCGAC TTTGTTACTTTATAGAAG | To generate the 3´ LTR of Piggybac transposon element |
| 3-LTR-R | cagc AAGCTT GGTACC ACCGGT CCTAGG TTAACCCTAGAAAGATAATC |
| LA-F2 | GAATTC GGACCGAAGCTGGCCAAGCCTCC | To detect the 5´ junction of targeted BLG locus in MDBK cells |
| MKR | GGGCTATGAACTAATGACCCCG |
| polyA-F1 | CCGCGACTCTAGATCATAATCAGCC | To detect the 3´ junction of targeted BLG locus in MDBK cells |
| RA-R2 | CCGCGG ACGGCAGTGTCTTCATCACCACC |
| M13F | ACG TTG TAA AAC GAC GGC CAG | TA cloning sequencing |
| M13R | CAG GAA ACA GCT ATG ACC ATG |
| T1-JK-F1 | GGCAAAGGGGTGCAAACCTAGC | To use with MKR for the detection of 5´ junction of MSTN locus replacement by IGF-I |
| EGFP-QF1 | TGAACCGCATCGAGCTGAAGGG | To detect the 3´ junction of MSTN targeting |
| TALEN-F3 | CTCTGGACATCGTACTGATC |
| Fw-miR222-PR | 5´-CGC AGATCTCCAAGCTAAACCCTTGCCTCACTG-3´ | Primers for pGL3-micro222-PR plasmid |
| Rv-miR222-PR | 5´-GCG AAGCTTCTACACATTCCAGCAGCTAC -3´ |
| Fw-mef2c | 5`- CCCAAGCTTATGGGGAGAAAAAAGATTCA-3` | Primers for construction of pcDNA3.1-MEF2C expression vector |
| Rv-mef2c | 5`- CCGCTCGAGTCATGTTGCCCATCCTTCAG-3` |
| mut-Fw-miR222-PR1 | 5`-TGTagtccgtacggTATGAATGCTCATATGAATGCTTTATTT-3` | Primers for direct mutagenesis of miR222 plasmid |
| mut-Rv-miR222-PR1 | 5`-ATAccgtacggactACACACTATATATGTATAACTGATTCACTTTGC-3` |
| mut-Fw-miR222-PR2 | 5`-CcccaagcccgtttaaGCATTTTAGGCAGTGCCAAAA-3` |
| mut-Rv-miR222-PR2 | 5`-CttaaacgggcttgggGCAAAGTAGGAAAAAAGATTGCTTG-3` |
| mut-Fw-miR222-PR3 | 5`-TTcccggggtaagCCCTTCTCTTTCCCTAAGGGC-3` |
| mut-Rv-miR222-PR3 | 5`-AGGGcttaccccgggAAGCGAAGTAGGTTTTTGTTTGATAC-3` |
| mut-Fw-miR222-PR4 | 5`-aacaagccggcccgtctcaCTTCTTGGAGATACCAGGCCTTT-3` |
| mut-Rv-miR222-PR4 | 5`-agacgggccggcttgttAGGGCCGCTGTAGCCAAT-3` |
| miR222 site A Fwd | CCAAGCTAAACCCTTGCCTCACTG | To clone site A and B of miR222 promoter for CHIP |
| miR222 site A Rev | GATTCACTTTGCTGAACACC |
| miR222 site B Fwd | GGTGTTCAGCAAAGTGAATC |
| miR222 site B Rev | CTATGAAGTATATTTTGGCACTGCC |
| Bio-WT-probe | 5`-GTGTGTTCTATAAATACTTCATTT-3`+Biotin | Probes for EMSA |
| Bio-Mut-probe | 5`- GTGTGTTCgtttcactgcaCATTT-3`+Biotin |
| WT-probe | 5`-GTGTGTTCTATAAATACTTCATTT-3` |
| Fw-ChIP-PCR-sideA | 5`-CCAAGCTAAACCCTTGCCTCACTG-3` | Primers for ChIP |
| Rv-ChIP-PCR-sideA | 5`-GATTCACTTTGCTGAACACC-3` |
| Fw-ChIP-PCR-sideB | 5`-GGTGTTCAGCAAAGTGAATC-3` |
| Rv-ChIP-PCR-sideB | 5`-CTATGAAGTATATTTTGGCACTGCC-3` |
| miR-222-RT-stemloop | GTCGTATCCAGTGCAGTGCAGGGTTCCGAGGTATTGCACTGCA  CTGGAT ACGAC ACCCAGAG | miR-222 specific RT primer |
| Fw-miR222 | GCCGC AGCTA CATCT GGCTA CTGG | miR-222 qPCR primers |
| Rv-miR222 | GTGCAGGGTTCCGAGGT |
| U6-RT-primer | AACGCTTCACGAATTTGCGT | U6 specific RT primer |
| Fw-U6 | CTCGCTTCGGCAGCACA | U6 qPCR primers |
| Rv-U6 | AACGCTTCACGAATTTGCGT |
| Drosha-F | AAAGGCCCATCCTGACCGAC | Primers for Drosha mRNA |
| Drosha-R | ATGCTGTGCCTGATTCCCGT |
| Dicer-F | CGGAGGATGATGAGGAGGACG | Primers for Dicer mRNA |
| Dicer-R | GCTGCTGCTGATGTAAGCCA |
| Fw-SCD5 | CTGCTCTGGGCCTACTTCTG | SCD5 qPCR primer |
| Rv-SCD5 | CCTGGACACTCGAAGATGT |
| miR222 probe | DIG-ACCCAGTAGCCAGATGTAGCT | Probe for Northern blotting |
